# Preparation of Pepsin and the Good to Be Obtained from It

**Published:** 1878-12-20

**Authors:** Joseph Adolphus

**Affiliations:** Ga.


### SOUTHERN MEDICAL RECORD.

VOL. VIII. ATLANTA, GA., DEC. 20, 1878. NUMBER 12.

EDITORS:

THOMAS S. POWELL, M. D. W. T. GOLDSMITH, M. D. R. C. WORD, M. D.

*R. C. WORD, M. D., Managing Editor.*

SUBSCRIPTION—$2.00 PER ANNUM, IN ADVANCE.

☞ All Communications and Letters on Business connected with THE RECORD, for 1877 and 1878, must be addressed to the Managing Editor.

### *ORIGINAL AND SELECTED.*

### PREPARATION OF PEPSIN AND THE GOOD TO BE OBTAINED FROM IT.

BY JOS. ADOLPHUS, M. D., OF GA.

In 1872 I read an interesting paper on the preparation of Pepsin, by an American author whose name I have unfortunately forgotten. The simplicity of the process, the way of obtaining the ferment according to the manipulations of this method, so impressed my attention as to fasten itself on my memory.

The process is so simple and easy of execution as to permit the whole thing being performed in a parlor, and is as follows:

The mucous coat of the calf's stomach is carefully stripped from its other attachments, and is finely chopped. It is then macerated in water, slightly acidulated with c. p. hydrochloric acid of sp. gr. 1.17, at a temperature of between 80 or 90 deg. for several days, during the time being frequently stirred, so as to wash out all the soluble pepsin from the gastric glands. The whole is then strained through muslin, and the residue pressed or squeezed with the hands to force out as much of the fluid from the magma as is possible.

The second step in the operation is to prepare a saturated solution of common salt in cold water, of which a quantity equal in bulk to the above solution is added to it, and the whole put aside and at rest for 24 or 48 hours. The pepsin will soon separate from the mother liquor and float on the fluid, from which it can be taken by a common spoon or perforated skimmer; it is then dried by pressing it between paper. Pepsin in this condition resembles plate paper and parchment. It is next dried in a cool, dry place, free from dust, and when well air dried, nine times its weight of good well dried sugar of milk is added, with which it must be well and thoroughly triturated. Ten grains of this represent one of pepsin. I have found the pepsin immediately after its preparation, and before it is air dried, to be readily soluble in glycerine at temperature of the body in the proportion of 15 grs. to the third ounce.

I have twice prepared pepsin after this formula, which is much the cheapest yet that has come under my notice. If the sugar of milk triturate is kept in quantities of small bulk in well secured glass coppered bottles, it will in a dry climate keep for two years. In the South it will not keep six months, if the least exposed.

The author of this process, if I recollect aright, observed that one grain of this pepsin dissolved in a few ounces of water slightly acidulated with hydrochloric acid, will dissolve 500 grains of coagulatad albumen. The glycerine process of obtaining pepsin is, to my mind, the best, but not as easy as the above. The only objections to be urged accuses the glycerine of taking up mucous and other impurities along with the pepsin. This however, is reduced to a minimum if the tissue is well washed in soft water so as to remove the mucous, which can be largely effected with care. I have frequently prepared pepsin after this manner, which is as follows:

The mucous coat of the calf's stomach, obtained in the same way as the other process, is well washed in clear, lukewarm soft water to free it from mucous. It is then well dried between cotton cloths, to abstract all superfluous water and moisture. After this it is chopped fine, and placed in a wide mouth, well fitting glass stopper bottle, and covered with pure glycerine at 1000 F.; then the stopper is carefully replaced so as to exclude the air and moisture. The bottle must be kept in a warm place and frequently shaken. Three days' maceration is long enough. The glycerine is then decanted, and the magma, which has been well pressed, is replaced in the bottle, and half as much water as there was originally of glycerine, slightly acidulated with hydrochloric acid (about 3 drops to the fluid ounce) is poured on it and the bottle again placed in a warm place, and frequently shaken. Eight or ten hours are long enough for this last maceration. The fluid is decanted and the magma is pressed. After allowing time to settle, the fluid is carefully decanted from the dregs. The decanted fluid is then evaporated to one-fourth its bulk, and added to the glycerine first obtained. This will keep for several years. I have had as good results from it after three years as when first prepared. It should be kept in bottles of one or two ounce bulk, well stopped, so as to exclude the air.

If properly prepared, 10 drops of this will dissolve 250 grs. of coagulated albumen in water, slightly acidulated with hydrochloric acid.

Now for the use of Pepsin. Omitting dyspepsia and the long list of its kind in chronic affections, for the cure of which it has been so well applied, few have thought of turning its valuable properties to assistance of patients suffering from acute diseases, to weather the storm till convalescence comes around.

In acute diseases the safety of the patient, in a great measure, depends on the quantity and duration of resisting stamina the organic forces are able to present to breast the surging waves of the destroying process called disease. Comparatively brief indeed, is the time consumed by these acute diseases in making their march, but brief as it is, the whole organic life forces of the economy are being rapidly sapped in different ways, but notably in largely cutting off the supply of nutrition in different ways, and as a secondary result, the impairment of the integrity of the hytologic elements of those tissues which largely compose the organs most essential to life.

Not having the space to enlarge on this interesting topic, let us proceed to the practical facts.

In typhoid fever experience has taught me that the major per centage of deaths occur between the close of the second week and the middle of the third; just the time when inanition would tell the heaviest on the resisting forces of the economy. If we omit such accidents as hemorrhage, perforation and the like, which are few in comparison as a cause of death, in typhoid fever, with exhaustion, the case clearly presents the truth of my proposition.

I hold that it exercises its dynamic force on organic and constructive processes of the economy after it enters the blood; by this means it increases the dynamic forces of nutrition, and saves life by increasing the molicular energies between the nutritive juices and the elements of the tissues. There are abundant facts to prove this. Very frequently convalescence is defeated because the patient is too much exhausted to get well.

This spring I was asked by a medical friend to see with him an old man who was very ill with typhoid pneumonia. The main features of his case, aside from the lung trouble, was very great exhaustion. I suggested pepsin, and the wine of it, which was of recent make, and was proven good, was used. In 24 hours a decided change for the better was observed, which brightened every day till convalescence was completed.

Two years ago I attended a lady through an attack of pneumonia. Her recovery was very problematic, on account of the extreme debility she was in. As a last resort I gave her pepsin that I had prepared with glycerine. Immediate improvement, almost, was visible after the taking. A notable point in these cases is the increased demand for food these patients soon make after taking the pepsin.

It is evident to all straight thinking people, that anything which will increase the powers of nutrition and assimilation in people suffering from acute disease, will increase also their chances of recovering.

This line of reasoning and the practice founded on it, apply to sick children in a very pointed manner.

Appropriation of nutritive pabulum can alone fortify against the collapse of the life forces and the cessation of function.

In pneumonia the largest per centage of deaths occur during the latter part of the first week and the beginning of the second. Death takes place from heart debility.

Without stopping to investigate the remote causes of death in pneumonia, we simply enquire: will the intelligent administration of pepsin contribute any thing toward retarding or removing the proximate cause of death? I think it does, and this too, on the single principles already alluded to.

When there is rapid destruction of tissue, the strain falls heaviest on those organs that are most used in carrying on the functions of life; among these prominently stand the heart and respiratory.

This fact points sharply to children, and explains why death in them so frequently commences at the heart and lungs. In point of fact, the major part of instances in which death is supposed to commence at the brain in children, is no more than defective blood supply to the brain, either in quantity, from heart feebleness, or in quality, from lung defectiveness.

The abdominal affections of children being attended by great emaciation and exhaustion, the value of pepsin to them cannot be well over estimated. The period of the disease in which it is proper to use pepsin is at the time, or immediately after, the disease has reached its height. In typhoid fever this is true; in pneumonia, seeing how rapidly heart failure comes on, the acme is reached no doubt very early, often on the 3d day.

The conditions that call for stimulants also call for pepsin. While stimulants can spur the naked and jaded energies into quicker movements, pepsin communicates to the molecular elements of the tissues a higher impulse, and awakens in them a more exalted energy to take food and live.

The dose of pepsin in all acute diseases must necessarily be small, not to exceed one-eighth or one-fourth of a grain every two or three hours, and always in a weak solution of hydrochloric acid, which must be strictly chemically pure. It may be, though in an humble way, the principles and facts set forth in this paper are a new departure from the philosophy of the schools.

